# Integrating maternal depression care at primary private clinics in low-income settings in Pakistan: A secondary analysis

**DOI:** 10.3389/fgwh.2023.1091485

**Published:** 2023-04-06

**Authors:** Syeda Somyyah Owais, Ronnie D. Horner, Muhammad Amir Khan, Kelli Kenison, Janice C. Probst

**Affiliations:** ^1^ Centre for Healthcare Resilience and Implementation Science, Australian Institute of Health Innovation, Faculty of Medicine, Health and Human Sciences, Macquarie University, Sydney, NSW, Australia; ^2^ Department of Health Services Research & Administration, University of Nebraska Medical Center, Omaha, NE, United States; ^3^ Association for Social Development, Islamabad, Pakistan; ^4^ Department of Health Services Policy and Management, University of South Carolina, Columbia, SC, United States

**Keywords:** Pakistan, maternal depression, primary healthcare (PHC), process evaluation, implementation

## Abstract

**Introduction:**

The prevalence of depression among women in Pakistan ranges from 28% to 66%. There is a lack of structured mental healthcare provision at private primary care clinics in low-income urban settings in Pakistan. This study investigated the effectiveness and processes of a facility-based maternal depression intervention at private primary care clinics in low-income settings.

**Materials and methods:**

A mixed-methods study was conducted using secondary data from the intervention. Mothers were assessed for depression using the Patient Health Questionnaire-9 (PHQ-9). A total of 1,957 mothers (1,037 and 920 in the intervention and control arms, respectively) were retrieved for outcome measurements after 1 year of being registered. This study estimated the effectiveness of the depression intervention through cluster adjusted differences in the change in PHQ-9 scores between the baseline and the endpoint measurements for the intervention and control arms. Implementation was evaluated through emerging themes and codes from the framework analysis of 18 in-depth interview transcriptions of intervention participants.

**Results:**

Intervention mothers had a 3.06-point (95% CI: −3.46 to −2.67) reduction in their PHQ-9 score at the endpoint compared with their control counterparts. The process evaluation revealed that the integration of structured depression care was feasible at primary clinics in poor urban settings. It also revealed gaps in the public–private care linkage system and the need to improve referral systems.

**Conclusions:**

Intervening for depression care at primary care clinics can be effective in reducing maternal depression. Clinic assistants can be trained to identify and deliver key depression counseling messages. The study invites policymakers to seize an opportunity to implement a monitoring mechanism toward standard mental health care.

## Introduction

Clinical depression has been estimated to affect 8% to 10% women of childbearing age (1). The prevalence of depression among women of reproductive age in Pakistan ranges from 28.8% to 66.0% (2). Routine primary healthcare usually does not specifically address maternal mental health (3). About 70%–80% of the primary care in low-income urban localities is sought at private clinics because of the scarcity of resources (4), mismanagement, and inaccessibility of public healthcare facilities (5). A lack of active depression screening and care in these low-income areas exacerbates the consequences of poverty (6).

Community-based cognitive behavior therapy and other psychotherapeutic approaches have been effective in reducing depression in mothers from low- and middle-income settings (3, 4, 7, 8). However, these approaches require extensive staff training and demand lengthy sessions to ensure proper delivery of the therapy conduct. The required skill level and time for such therapies is infeasible for private primary care facilities in Pakistan, where a clinic assistant (CA) helps a doctor in providing general medical care to a high volume of patients.

To address these challenges, an alternative maternal depression counseling intervention was developed as a secondary component of an early child development (ECD) randomized control trial focusing on infants aged 0–12 months in low socioeconomic urban settlements in Pakistan (9). The primary component of the trial focused on educating mothers on activities that would enhance the brain development of their trial-registered infant (registered at <6 weeks old). This was a clinic-based care model where the mother would visit the clinic on a 3-monthly basis to receive instructions on activities to perform with their child at home to further their development (10). Child development was measured using the Ages and Stages Questionnaire-3 at endpoint, which was when the child reached 12 months of age. The effectiveness of the intervention was estimated by comparing the number of child development delays in the intervention and control arms. There were significant endpoint differences in the prevalence of delays in two or more child development domains (17%; 95% CI: −0.26 to −0.09; *p* = 0.001) (10). The primary study also reported that the proportion of mothers with depression in the control arm was 23% (95% CI: −0.29 to −0.18; *p* < 0.001) more than that in the intervention arm. However, this proportion only indicated a perfunctory difference, reflecting differences in average point-prevalence between the control and the intervention arms at endpoint; no baseline comparisons or change in depression scores were measured. Moreover, the process evaluation on the trial reported only on the primary intervention's—early child development—implementation process and did not report on the maternal depression intervention–specific processes and outcomes.

This study aimed to further explore the impact of the maternal depression intervention component by estimating the difference in change in depression scores between the baseline and the endpoint measurements for the trial arms through a secondary analysis of the collected data. Estimating the change in depression scores may help understand the magnitude of impact of such an intervention. The study also aimed to assess the implementation process of the maternal depression intervention component to gain insight into the experiences, feasibility, and fidelity of delivering a counseling package at primary care clinics in low-resource settings.

## Material and methods

### Study design

This mixed-methods (11) study was conducted using data collected for the maternal depression intervention component from the ECD trial at private primary care clinics in Pakistan (10). Baseline and endpoint PHQ-9 (Patient Health Questionnaire-9) scores were used to estimate the change in depression scores among mothers of the intervention and control arms. Semistructured interviews with the intervention participants were used to explore the implementation process of the depression intervention component. Access to deidentified data was provided for analysis for this study.

### Participants

The cluster randomized control trial (targeting mothers of 0–12-month-old children) was implemented at 32 private clinic clusters (randomized 16 in the intervention and control arms, each) in low socioeconomic urban settlements in two districts of Pakistan, Lahore and Rawalpindi. The participation of clinics was facilitated by the respective district health offices. Each clinic staff included a certified general medical practitioner and a CA. A CA is usually a local male, trained on-the-job by the clinic doctor, but is not a formally trained paramedic (9). The trial had aimed to measure the effectiveness of a facility-based maternal counseling package delivering key messages on child development, nutrition, and maternal depression.

After randomization of the clinic clusters, each clinic doctor and CA was trained on participation in the research, including recruiting mothers and recording demographic information. All clinic staff were briefed on maternal depression and the administration of PHQ-9. Intervention clinics were further informed about the designed pictorial flipbook counseling tool. Intervention CAs were trained on the use and conduct of the flipbook *via* roleplay. Intervention doctors were advised to confirm diagnosis, prescribe medication, or refer to tertiary care.

**Figure 1:**
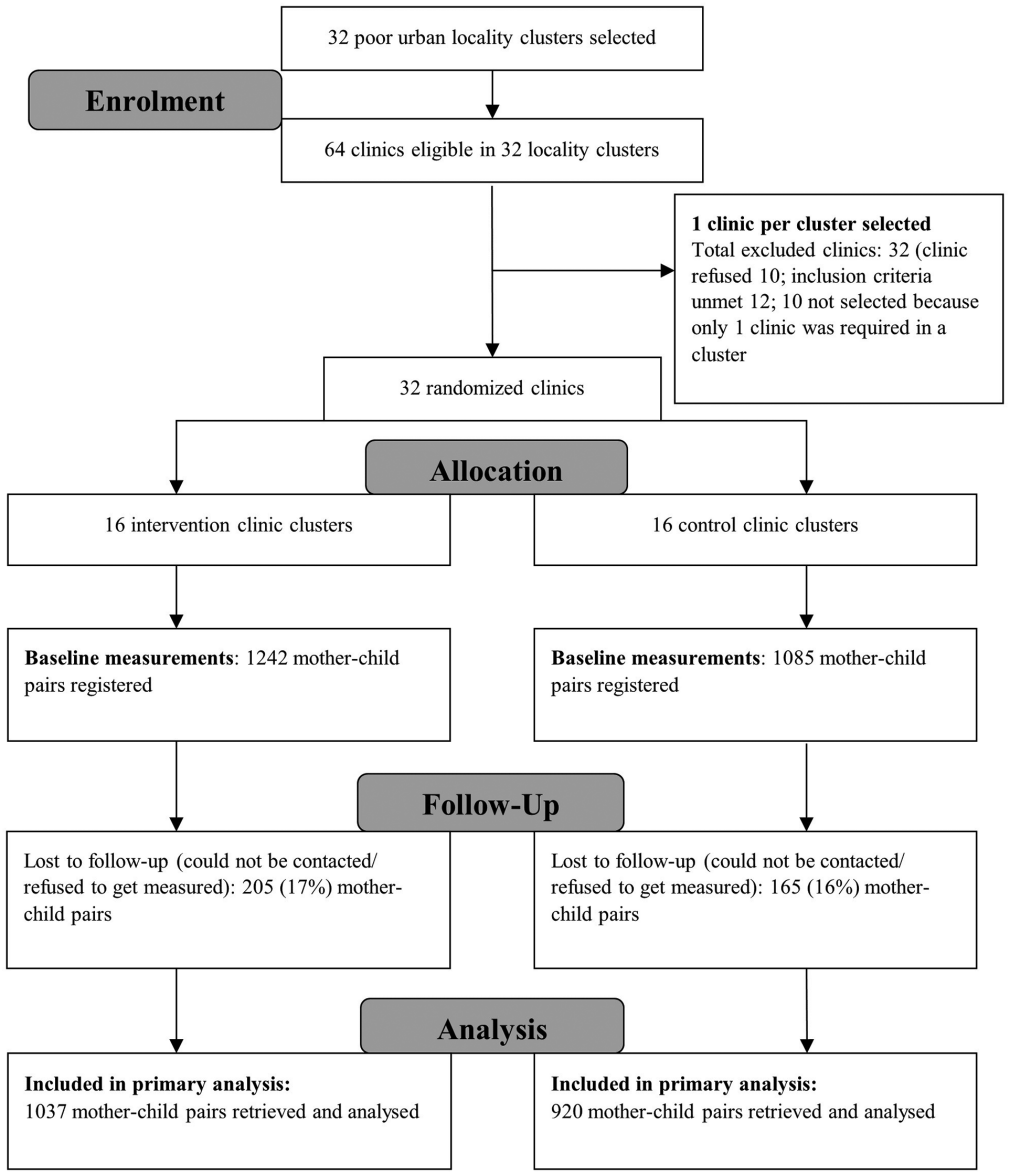
CONSORT flow diagram of ECD trial (0–12 months).* [*Adopted from published trial outcomes (10)]. ECD, early child development.

A total of 2,327 mothers of infants aged ≤6 weeks (intervention = 1,242; control = 1,085) were recruited between April 2014 and September 2016. Out of these, 1,957 mothers (intervention = 1,037, 83.5%; control = 920, 84.8%) were successfully followed up for outcome measurements after 1 year, when the registered child reached 12 months of age (Figure 1) (10).

### Procedure

**Figure 2:**
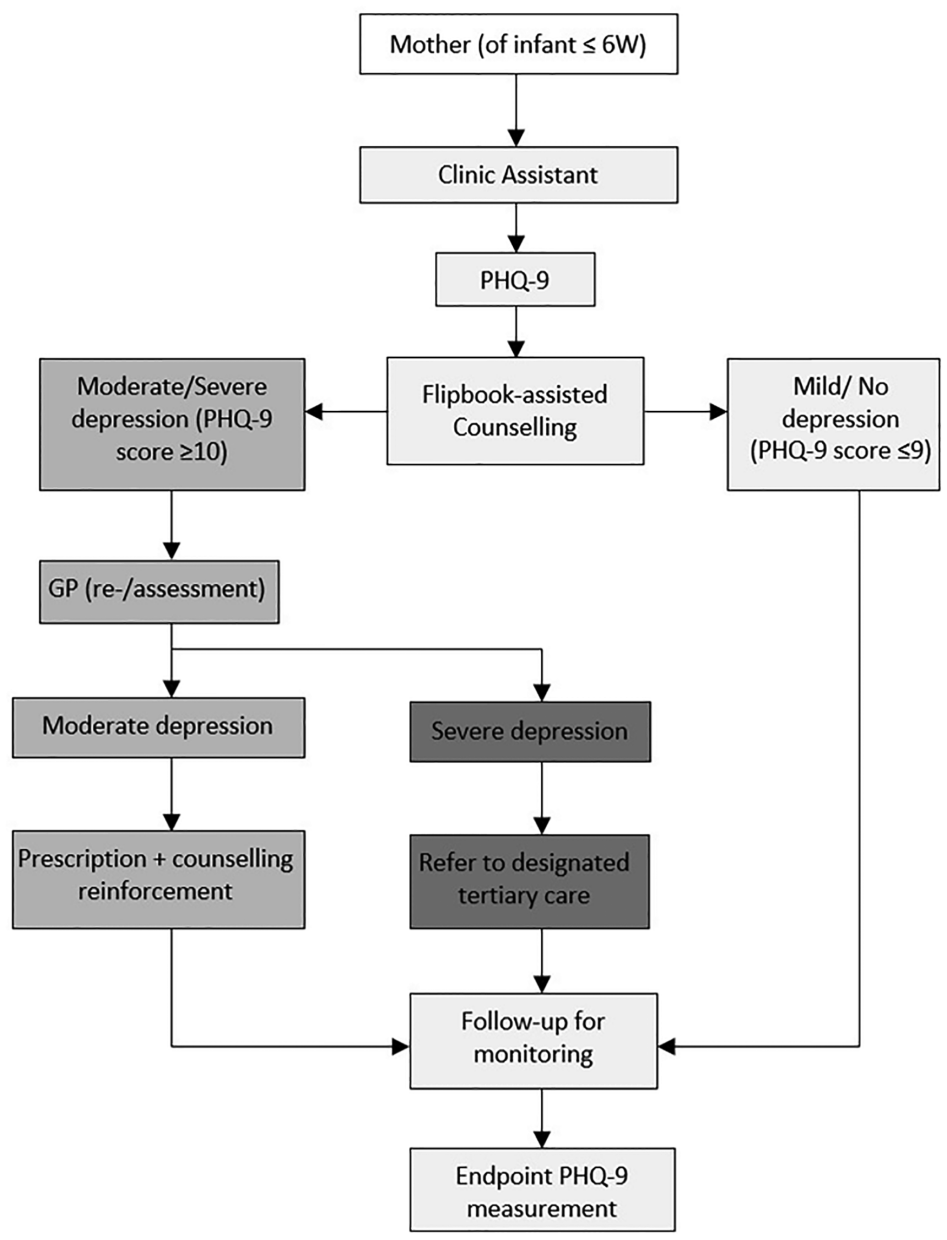
Depression intervention flow diagram.

The depression intervention component was developed for: (a) a CA to screen, counsel, and refer mothers for consultation to the clinic doctor at registration; (b) a primary care practitioner to diagnose, prescribe a designated antidepressant, and refer for specialist consultation (Figure 2). Mothers were counseled by the CA on a quarterly basis on depression prevention/management behaviors using a pictorial flipbook. Depression assessment was conducted by administering PHQ-9, a nine-item diagnostic tool continuously scored from 0 to 27 with score intervals indicating depression severity (12).

The CA screened mothers for depression using PHQ-9 at registration. All mothers were counseled using the six behavior activation pictures in the flipbook, regardless of their depression score. The pictures were aided by prompts from the CA. The six behavior activations were meditation (mental relaxation), sleeping or resting (physical relaxation), participating in personal care and grooming (to enhance self-esteem), indulging in pleasure/leisure activities (e.g., watching television, reading a book), venting out feelings with a trusted friend, and socializing with friends or family. Mothers were then advised to come for a follow-up after every 3 months for a reassessment.

Following the counseling at registration, mothers with a PHQ score greater than 10 were sent to the clinic doctor, who verified the scoring. Mothers with moderate depression were prescribed an antidepressant and were asked to visit after a month for monitoring purposes. Mothers with mild and moderate depression were reassessed on each follow-up visit. The doctor referred mothers diagnosed with severe depression for specialist care at an identified specialist tertiary care facility.

Control clinic staff were instructed to record registered mothers’ PHQ-9 and provide care as per their usual clinical practice.

Trial endpoint measurements for each mother's depression (i.e., when registered child was 12 months old) was recorded by recruited “blinded” assessors using PHQ-9.

**Figure 3:**
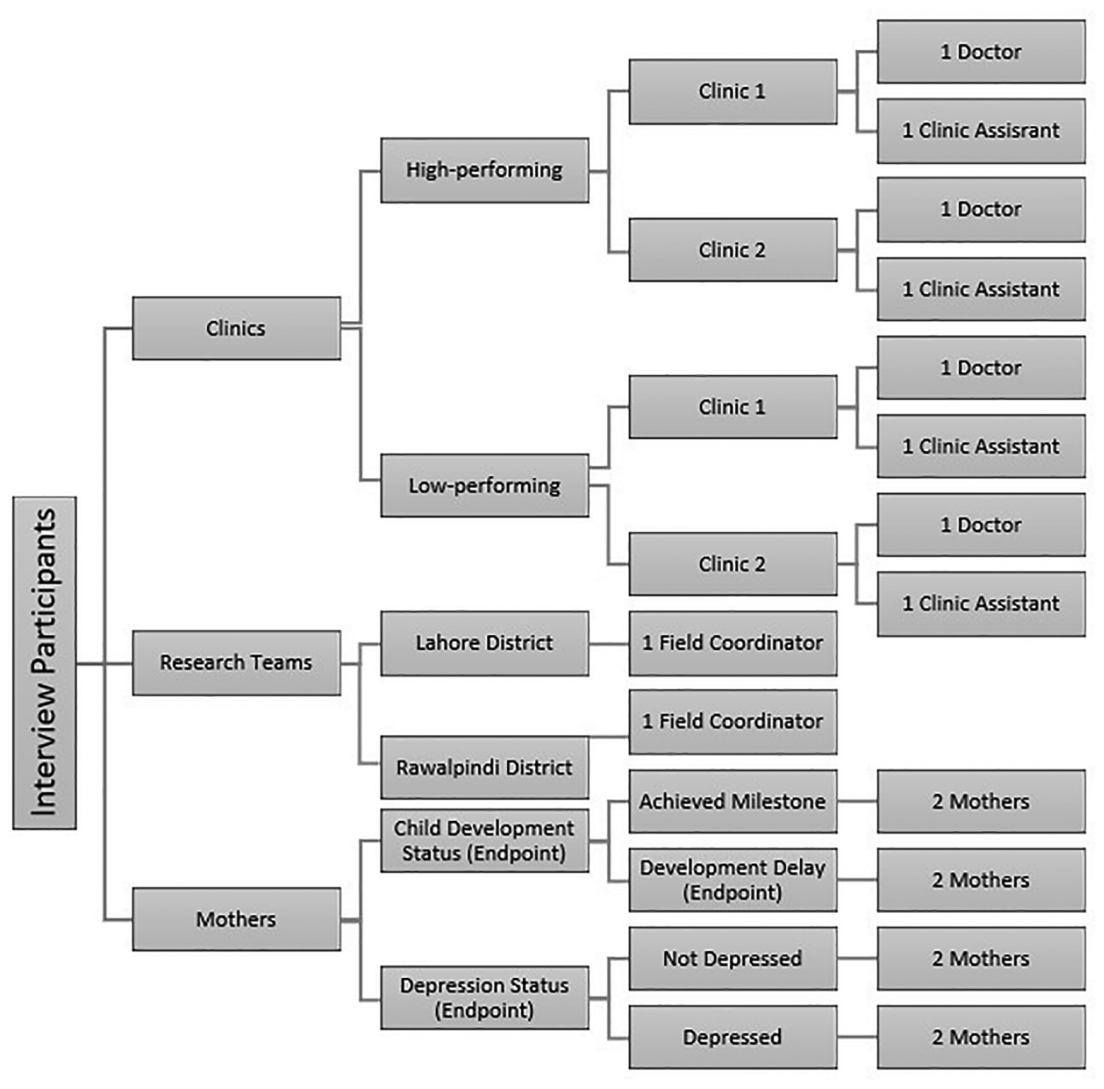
Illustrative description of interview participants [adapted from the primary outcomes process evaluation (13)].

A total of 18 semistructured interviews with participants in the intervention arm were conducted by trained qualitative researchers using a developed evaluation framework and guide. The evaluation framework and guide were developed with a team of experts to explore various processes and stages within the intervention course. These included, but were not limited to, prompts about approaching public engagement for the implementation of the intervention at clinics; engagement, training, and onboarding of clinic staff; care provision of mothers; monitoring and evaluation of care delivery and data at clinics and outcome measurement (Supplementary Appendix S1). The framework was used to inform the process evaluation for the primary outcome of trial (13). Interview participants were selected from clinics categorized into high and low performance on the basis of the volume of mothers registered, client adherence to follow-up visits, and facility performance indicators (e.g., record keeping). One facility per performance category per district was then randomly selected for qualitative data collection. Two healthcare providers and two mothers (arriving for the endpoint measurement) were interviewed at each of the four selected facilities. Two researchers on the trial field implementation team were also interviewed (Figure 3). Each 30-min interview was conducted in the local language (Urdu) and audio-recorded. Some respondents were also subsequently called by telephone to clarify or elaborate on their initial responses. The trial research team transcribed the audio recording and translated it into English. The translated transcriptions were provided for the current analysis.

### Outcome

The study measured the difference in the change in PHQ-9 scores, ΔPHQ-9, between the baseline and the endpoint mothers of the intervention and control arms. ΔPHQ-9 was calculated by subtracting each mother's endpoint PHQ-9 score from her baseline PHQ-9 score. Equation (1) details this calculation, whereby ΔPHQ-9_jki_ is the change in PHQ-9 score for each mother *i* in *j* clinic in *k* arm, i.e., either intervention or control:

(1) $$\Delta \mathrm{PHQ}\text{-}9_{\;jki}=\mathrm{PHQ}\text{-}9b_{\;jki}\text{–}\mathrm{PHQ}\text{-}9e_{\;jki}$$

To maintain methodological consistency and reporting, the qualitative outcomes were assessed using the same predefined domains indicated by the process evaluation framework: organizational (provider and community engagement), case management (care delivery by clinic staff), and continuity of care (13).

### Data analysis

A quantitative analysis for the outcome compared ΔPHQ-9 for obtaining individual-level data on the mothers of the intervention and control arms to estimate the significance of the intervention effect through multilevel modeling, i.e., ΔPHQ-9 (14) A mixed-effects model was used to assess the significance of difference in the change in PHQ-9 scores for the trial arms: ΔPHQ-9 and covariates were fixed effects, and clinic clusters were random effects in Stata IC (version 15.1), detailed by the basic model (2) below, whereby $y_{ij}$ indicates the ΔPHQ-9 score, and $\beta_{0}$ indicates the mean ΔPHQ-9 score for the control arm clusters, whereas $\beta_{1}$ is the estimate for the intervention arm effect adjusted for all the covariates, $\beta_{n}$, for the mothers, $x_{ij}$. The random effect of clinic clusters *j* across all mothers is denoted by $\alpha_{j}$.

(2) $$y_{ij}=\beta_{0}+\;\beta_{1}x_{ij}+\;\beta_{n}x_{ij}+\alpha_{j}+\;\varepsilon_{ij}$$

The analysis controlled for covariates collected at baseline: education (years of schooling), mother's age (years), family status (living joint vs. nuclear family), total number of children, number of children below 5 years of age, child's sex, baseline measurement for stunting (below 2 SD height for age), and weight for age (below 2 SD). To control for effects from other intervention components, the change in the PHQ-9 scores was also adjusted for endpoint measurements of child development, i.e., the number of delays in development categories according to Ages and Stages Questionnaire-3, stunting, and weight for age.

A qualitative analysis was conducted by using SSO and cross-checked by using KK employing the framework method, whereby the authors charted the data and codes into the predeveloped framework matrix: organizational (provider and community engagement), case management (care delivery by clinic staff), and continuity of care (15). The analysis was an iterative process by which both researchers read each transcript multiple times and identified and compared the initial codes. Comparisons were made *via* weekly meetings in which both researchers discussed the similarities and differences in their codes along with their understanding of each transcription, after which final codes were developed for each respondent category, i.e., doctors, CA, research staff, and mothers. The final 57 codes were organized under the domains from the predefined framework.

A *post-hoc* analysis was also conducted to measure whether the change in the PHQ-9 scores for mothers resulted in a change in depression category as measured by PHQ-9. A mixed-effects ordered logistic model was used to estimate the predicted proportional odds of whether the change in the PHQ-9 scores resulted in an improvement in mothers’ depression status (a change from either moderate to mild/no depression, or mild to no depression), a deterioration (a change from either no depression to mild/moderate depression or mild to moderate), or no change (no change in depression status as per PHQ-9 categories), denoted by the model (3) below:

(3) $$\log\left[\frac{P_{ijc}}{1-P_{ijc}}\right]=\gamma_{c}-\;[x_{ij}\beta +v_{j}]$$

The cumulative probabilities of the three ΔPHQ-9 categories are denoted by $P_{ijc}$  = Pr(Y_ij _≤ c), where Y_ij_ represents the ΔPHQ-9 category for a mother, *i*, in the *j*th clinic cluster. *c* represents the categories within the ΔPHQ-9 category variable. It is important to note that the ordinal logistic regression model estimates probabilities with thresholds for each category, $\gamma_{c}$, within the outcome variable. Model covariates are represented by $x_{ij}$ with β regression slopes. The clustering effect is measured by using $v_{j}$ as a random effect, whereas the coefficients β and thresholds $\gamma_{c}$ are fixed parameters.

## Results

This study aimed at evaluating the effectiveness and implementation of a maternal depression intervention, a secondary component of a cluster randomized control trial, at private clinics in low socioeconomic urban localities in Pakistan. Of the 2,327 enrolled mother–child pairs, 1,957 (intervention = 1,035; control = 920) were retrieved for the endpoint measurements to record the mothers’ PHQ-9 scores and child outcomes (10).

Table 1 presents the characteristics of mothers retrieved for endpoint measurement. About 70% of mothers were recorded to have no depression at baseline for both arms. The endpoint measurements indicate that the intervention arm children had a fewer number of development delays (about half the number of delays compared with control). The proportion of children with stunting and reduced weight for age was also less compared with control (13% compared with 32% and 22% compared with 30%, respectively).

**Table 1:** Distribution of characteristics for maternal depression intervention at private clinics (*N* = 1,957, intervention = 1,037, control = 920).

Mean (SD)		Control	Intervention
Cluster size		57.5 (50.9)	64.8 (42.3)
Baseline^a^ child age (days)		10.31 (14.6)	16.2 (14.5)
Total children		2.67 (1.24)	2.60 (1.37)
No. children under 5 years		1.65 (0.61)	1.60 (0.60)
Mother age		27.1 (3.16)	27.2 (3.45)
Mother education		8.61 (3.80)	8.30 (3.97)
Endpoint^b^ no. of delays^c^		1.07 (1.06)	0.57 (0.80)^d^
*N* (%)			
Child sex	Male	486 (52.8)	572 (44.8)
	Female	434 (47.2)	465 (55.2)
Family status	Nuclear	226 (24.6)	213 (20.5)
	Joint	694 (75.4)	824 (79.5)
Baseline child stunting^e^	Normal	689 (75.0)	777 (74.9)
	Stunted	231 (25.0)	260 (25.1)
Baseline child weight for age^f^	Normal	661 (71.8)	735 (70.9)
	Underweight	259 (28.2)	302 (29.1)
Endpoint child stunting	Normal	624 (67.8)	903 (87.1)^d^
	Stunted	296 (32.2)	134 (12.9)^d^
Endpoint child weight for age	Normal	643 (69.9)	805 (77.6)^d^
	Underweight	277 (30.1)	232 (22.4)^d^
Baseline maternal depression^g^	No depression	669 (72.7)	761 (73.4)
	Mild	140 (15.2)	126 (12.2)
	Moderate	110 (12.0)	146 (14.1)
	Severe	1 (0.10)	4 (0.40)

^a^ Baseline measurements for the child were made when the child was ≤6 weeks of age when the mother brought the child to be registered in the trial.

^b^ Endpoint measurements were made when the child was 12 months old.

^c^ Number of delays was categorized by the number of development categories in which the child was found to be delayed at the 12th month of age according to Ages and Stages Question-3.

^d^ Significant child endpoint difference recorded, *p* < 0.01 (10).

^e^ Stunting was below 2 SDs height for age; above the given cutoff was termed “normal” height for age.

^f^ Underweight was measured below 2 SDs weight for age; above the given cutoff was termed “normal” weight for age. WHO age-appropriate growth charts were used to determine cutoff and assessments.

^g^ Mothers were assessed for depression categories according to Patient Health Questionnaire-9. All baseline measurements were conducted by clinic staff.

The quantitative analysis, i.e., the mixed-effects model (Table 2) shows that mothers in the intervention clinics had an average of 3.19-point (95% CI: −3.61 to −2.77; *p* < 0.001) reduction in PHQ-9 scores at endpoint compared with mothers in the control clusters. This difference in the reduction of PHQ-9 scores from baseline to endpoint was significant even after adjusting for covariates and confounding by child development intervention; mothers in the intervention clusters had an average of 3.06-point (95% CI: −3.46 to −2.67; *p* < 0.001) reduction in PHQ-9 scores compared with the control arm.

**Table 2:** Change in PHQ-9 scores for mothers after 12 months (*N* = 1,957, intervention = 1,037, control = 920).

	Crude model	Adjusted model
	Estimate (95% CI)	Estimate (95% CI)
Intervention effect^a^ ΔPHQ-9	−3.19 (−3.61 to −2.77)^b^	−3.06 (−3.46 to −2.67)^b^
Mother age (years)	—	−0.03 (−0.08 to 0.03)
Mother education (years)	—	0.03 (−0.01 to 0.07)
Total children	—	0.11 (−0·05 to 0.28)
No. children under 5 years	—	−0.26 (−0·55 to 0·03)
Child gender		
Female	—	Ref
Male	—	0.04 (−0.26 to 0.35)
Baseline^c^ child age (days)	—	0.01 (−0.01 to −0.02)
Baseline child height for age		
Not stunted	—	Ref
Stunted^d^	—	−0.05 (−0.47 to 0.37)
Baseline child weight for age^e^		
Standard	—	Ref
Underweight		0.02 (−0.40 to 0.45)
Family System		
Nuclear	—	Ref
Joint	—	0.31 (−0.07 to 0.70)
Endpoint^f^ child height for age		
Standard	—	Ref
Stunted	—	−0.02 (−0.65 to 0.14)
Endpoint child weight for age		
Standard	—	Ref
Underweight	—	0.03 (−0.34 to 0.39)
Endpoint no. of delays^g^	—	0.46 (0.28 to 0.63)^b^
ICC	0.09	0.07

ICC, intraclass correlation coefficient.

^a^ Compared with the control arm.

^b^ *p* < 0.001.

^c^ Baseline measurements for the child were made when the child was ≤6 weeks of age when the mother brought the child to be registered in the trial.

^d^ Stunting was below 2 SDs height for age; above the given cutoff was termed “normal” height for age.

^e^ Underweight was measured below 2 SDs weight for age; above the given cutoff was termed “normal” weight for age.

^f^ Endpoint measurements were made when the child was 12 months old.

^g^ Number of delays was categorized by the number of development categories in which the child was found to be delayed at the 12th month of age according to Ages and Stages Question-3.

The *post-hoc* analysis revealed that intervention clinic mothers had a 27% (95% CI: 0.13–0.40; *p* < 0.001) higher odds ratio of improvement in their depression status (PHQ-9 category) than no change or deterioration compared with a 2% (95% CI: 0.004–0.04; *p* = 0.018) odds of improvement from the usual care practiced in the control arm (Supplementary Appendix S2).

Emerging themes from the qualitative analysis were allocated to relevant categories in the predefined framework described below.

### Organizational

#### Provider engagement and public–private partnership

Care model development was focused on being culturally adaptable and acceptable for successful integration within private clinic workflow. The research team emphasized the cultural relatability of pictorial messages so that doctors and CAs could easily counsel mothers.

“… we had focus group discussions with the mothers from similar backgrounds to help us understand the daily routine and problems… experts assessed if the picture messages were imparting what we were trying to say…” (research staff).

Even though the innovativeness of the intervention motivated clinics to participate in the research, district health office endorsement of the service created a marketing opportunity to attract clients. Clinics were able to provide the service at no extra cost to the existing consultation. The district health office collaboration helped identify urban communities with the lowest socioeconomic indicators. Posters and brochures with district office endorsement helped promote uptake.

“… getting the district health on board was great… reflected that quality measures were taken by the government” (doctor).

Clinic staff training prior to commencement of the trial was conducted by psychology/psychiatry specialists so that specific questions could be addressed. CAs were trained using the roleplay of scenarios to address questions mothers could possibly have during the counseling session. Roleplay was especially appreciated as a training method. Doctors were receptive of the training and asserted that they were aware of depression diagnostic guidelines but were passive about screening mothers unless they explicitly asked for help.

#### Community advocates engagement

Central grocery stores, drug stores, and mosques were selected for intervention promotion. However, mobilization as community advocates was challenging because of the lack of existing data. Volunteer vaccinators (through the government's Expanded Program of Immunization) were also recruited in consultation with the district health office; however, establishing a referral of clients to clinics was challenging because of the lack of private-to-public (and vice versa) link to care. Nonetheless, most mothers seemed to have been referred by a vaccinator.

### Case management

#### Care uptake and assessment

Mothers expressed a greater interest in learning about their child's development than about their own mental health. They willingly answered the PHQ-9 questions asked by the CA; however, mothers accompanied by someone (husband/mother-in-law) showed hesitation during the assessment.

“I had to tell them (mothers) that this is something new that we were introducing at the clinic and if they are uncomfortable answering, they can choose not to answer (PHQ), no one refused” (clinic assistant).

#### Care delivery

Being counseled by a male CA was acceptable; mothers reflected that they were able to discuss minor problems with CAs related to the prescribed mental health management behaviors. The flipbook pictures were found to be comprehensive; mothers were able to understand the messages with minimal prompts. This helped CAs deliver the counseling within the time constraints. Mothers were able to practice the behavioral management activities at home; however, some claimed it was difficult to find time to do so while managing other responsibilities.

“… I was very good at guessing what the pictures were saying … he gave me a summary and told me why these were important, and I agreed” (mother).

Doctors found that many mothers failed to report their symptoms if they were not assessed using PHQ-9. For these mothers (who were unaware of linked mood disturbances), doctors had to inform them about their depression diagnosis delicately, cognizant of depression-related stigma. Most mothers understood their symptoms and diagnosis and were willing to be prescribed medication for management. However, some expressed reluctance about informing family members of the specific reason for the prescription. The prescribed medication was reported to be affordable, but doctors were unable to ensure whether mothers were adhering to their advised prescription.

“I couldn't tell mother-in-law it (medication) was for depression, then others would find out and think all sorts of things … it makes me uncomfortable” (mother).

### Continuity of care

#### Follow-up visits

Contacting most mothers for their follow-up was challenging because they registered their husband's contact numbers and it was unknown whether the follow-up reminder was passed on. Another challenge was convincing mothers to attend, because they would seldom feel the need for a follow-up due to the absence of a physical ailment. Mothers preferred visiting for their follow-up according to their time availability and were less adherent to the given follow-up schedule.

#### Referral

A few mothers diagnosed with severe depression were referred to the designated specialist; however, it was beyond the clinic systems to ensure or keep a record of whether these mothers had sought treatment. The clinic staff could not go beyond the recommendation of referral because of the fragmented private-to-public healthcare linkage.

“We referred one to the specialist, but it was hard to convince her … no way to tell if she went …” (clinic assistant).

#### Monitoring and evaluation

The monitoring and evaluation assimilated experiences of clinic performance related to intervention delivery. Research field staff conducted fortnightly evaluation visits to each clinic. The intermittent involvement of the district health officers during these visits helped in aligning clinic outputs to intervention goals.

## Discussion

The purpose of this study was to evaluate the effectiveness and implementation process of a maternal depression intervention component at private primary care clinics in low socioeconomic urban communities in two districts of Pakistan. There was a significant reduction in the PHQ-9 depression scores for mothers who visited the intervention clinics compared with control, −3.06 (95% CI: −3.46 to −2.67). Mothers of the intervention clinic clusters also had higher odds of experiencing an improvement in their PHQ-9 depression category compared with the control arm, 27% (95% CI: 0.13–0.40; *p* < 0.001) vs. 2.3% (95% CI: 0.004–0.04; *p* = 0.018).

A strength of this study is that it goes a step forward by analyzing point-score differences in each mother's PHQ-9 score—this answers a vital question of whether depression was indeed reduced or prevented in mothers in the intervention arm, where improvement was intended. We were able to determine that there was, in fact, an average of 3.06 point-score reduction of PHQ-9 scores for mothers in the intervention arm. But what did this mean? This warranted the *post-hoc* analysis through which we determined whether the change in score for mothers resulted in a change in their depression category according to the PHQ-9 classification: no depression, mild, moderate, and severe depression. This provided us a way to truly determine whether a reduction in score meant that a mother who was initially depressed at baseline experienced improvement in her symptoms.

The reduction in the depression scores suggests that a behavioral activation intervention, delivered by an on-the-job trained CA, can help manage depression symptoms in mothers visiting private primary care clinics. Similar interventions have been implemented in Pakistan, but with little promise of sustainability; these interventions focused on door-to-door identification and counseling of mothers rather than integrating a structured facility-based model. Moreover, they either focused on extensive staff training or were unable to substantiate evidence through the use of a comparable control group (16–18). The intervention analyzed in this study emphasizes that a simple intervention, catered to existing clinic structures and model of care, can have similar, if not equal, effects of reducing depression and can also be more effective in terms of providing sustainable mental healthcare.

The use of a structured depression tool to screen or measure depression helped identify cases that could have otherwise been missed opportunities—this should be seen as a crucial indicator for policymakers for closing any gaps in care. The brief counseling session and simpler content made it feasible for clinic staff to incorporate mental health counseling into routine practice. It was also feasible for mothers because the behavior activations were developed to require minimal disruption for them. However, discomfort associated with depression diagnosis and related medication prescription (especially in the presence of family members) indicated the presence of mental health–related stigma. Low follow-up adherence indicates a low perceived need to visit a health facility in the absence of a physical ailment. This implies a lack of recognition or priority of mental health needs among women in low-income urban Pakistan. The perception that mental health difficulties are not priority health problems is a desperate cry toward improving healthcare goals (19). It highlights the lack of acceptability of mental health conditions among communities in developing countries. Furthermore, it indicates not only the lack of awareness education but also the lack of promoting the need to seek mental healthcare. Evidence suggests that interventions for mental health promotion involving support from family, community leaders, and religious belief systems could improve effectiveness (16, 20, 21). Enabling acceptance of mental health issues may aid mothers in communicating these problems with their families in an effective manner. This may lead to family members contributing toward the improvement of depression symptoms and providing added emotional and financial help in seeking appropriate care.

Although the process evaluation gave an insight into the need for the integration of depression-related services at the primary care level, it also indicated key opportunities for improvement in the system of providing better mental health services. These include depression awareness campaigns and setting up linkage systems for better identification and referral of cases. Involvement of the respective district health offices and communities allowed a better engagement of private service providers and care uptake. Outreach and sustainability have proved more successful through the engagement of stakeholders, community leaders, and policymakers during the implementation process (22, 23).

An important observation was that clinic doctors had the discretion of charging an extra fee for the “new” integrated care being provided, however, most did not. Uptake could have been influenced by the fact that the care came at no extra cost to the mothers. Potential future implementation of an extra fees for depression care could be investigated as predictors of uptake and care provision. This extensive evaluation of the depression intervention is unique to Pakistan, which makes it difficult to compare other strategies used for depression intervention in Pakistan.

An inherent limitation in a facility-based intervention is that the benefits of the care are utilized by those who visit the facility as opposed to those who do not for several reasons, including, a lack of affordability and means for traveling, cultural or domestic restraints/restrictions, and a lack of awareness. The effectiveness (reduction in depression among mothers of the intervention arm) cannot be exclusively attributed to the maternal depression intervention component alone; it could be a combined effect from the childcare components that enhanced child nutrition and brain development. Nonetheless, any potential confounding was addressed by adjusting for child-related outcomes in all analyses.

The interview responses by participants could have reflected positive intervention effects to meet self-inferred expectations; there was also a lack of knowledge about the household circumstances in which the mothers practiced their behaviors. Being a secondary data analysis, interviews had already been conducted and these limitations could not be excluded.

## Conclusion

The study evaluated an effective depression management care package that was integrated within private clinic work settings to cater to the mental health needs of mothers in low socioeconomic urban communities where affordable quality mental healthcare is often out of reach. It offers avenues to bridge broken care linkages between private primary care and tertiary care in the public sector, especially in the area of mental health.

## Data Availability

The data analyzed in this study is subject to the following licenses/restrictions: Data requests can be sent to the principal investigator, coauthoring on this study, for consideration. Requests to access these datasets should be directed to MAK, Association for Social Development, ccp@asd.com.pk.
