# Supplementary material for: Integrating maternal depression care at primary private clinics in low-income settings in Pakistan: A secondary analysis
Source: Front Glob Womens Health. 2023 Apr 6;4:1091485. doi: 10.3389/fgwh.2023.1091485 (PMC10117980; doi:10.3389/fgwh.2023.1091485)
Supplement: Supplementary file 1 [file Table1.docx]

**Early Child Development Care Delivery through Private Clinics in Pakistan**

**Trial Process Evaluation**

**Tool-1: Interview Guidelines – Facility Doctor**

| **Query** | **Prompts and Probes** |
| --- | --- |
| **Preparation for Care Delivery Process** | |
| Would you kindly share you experience of the initial **survey** for the **selection** of your clinic in the ECD care delivery? | - Experience of your getting informed and participate in the survey for mapping/ selection of clinic (include information content, why cooperated)?   - *Probes:*     - What (if any) queries did you dislike? and how managed?     - Selection of clinic transparent? why? |
| Would you kindly share your experiences of **District Health Office engagement** in care delivery? | - Experience of the district health office engagement in enabling of private clinics for ECD care (include challenges and how managed)?   - *Probes:*     - Helpful or Not; and why? |
| Would you kindly share your experiences regarding the agreed **branding/promotion support**? | - Expectation of your branding (style and form)?   - *Probes:*     - Branding structure and contents?     - Why has there been a deviation, if any, from the expected branding? - Experience of the actual clinic branding structure and contents (include challenges and how managed)?   - *Probes:*     - Structure – feasibility, challenges?     - Contents – clarity, visibility, acceptability? |
| Would you kindly share your experience regarding the **training** of **Early Child Development Care**? | - Experience of your getting trained on ECD care (include challenges and how managed)?   - *Probes:*     - Selected time and venue?     - Training contents – language, methods, duration, practical relevance etc?     - Trainer’s – knowledge and skill, facilitation ability,     - Arrangements – seating, cooling or heating, refreshment, time-keeping, house-keeping etc?     - Providing feedback on training conduct? |
| Would you kindly share your experiences of **engaging and working with community advocates**? | - Experience of engaging community advocates for ECD care promotion (include challenges and how managed)?   - *Probes:*     - Various types of community advocates?     - Referral arrangements for ECD care?     - Suggestion (if any) for future scaling of community advocates? |
| **Delivering the Intervention** | |
| Would you kindly share your experiences of **case examination and registration**? | - Experience of registering and examining child for ECD care (include challenges and how managed)?   - *Probes:*     - Educating mother to get her child registered for ECD care?     - Measuring the weight and length of child?     - Asking for quarterly clinic visit |
| Would you kindly share your experiences of **assessing and managing mothers’ depression**? | - Experience of screening and counselling mother for mental health (include challenges and how managed)?   - *Probes:*     - Administering PHQ-2? Mother responses?     - Administering counselling? - Experience of diagnosing and managing depression in mothers (include challenges and how managed)?   - *Probes:*     - Administering all PHQ-9 questions? Mother responses?     - Prescribing anti-depressant?     - Referring for specialist care (severe depression)? |
| Would you kindly share your experiences of **maintaining records** for ECD care? | - Experience of keeping individual case records contrary to usual practice (include challenges and how managed)?   - *Probes*     - Logistics – staff time, space, clinic routine?     - potential benefit (e.g. PHC certification)?     - any risk (e.g. legal, tax) of maintaining case records? |
| Would you kindly share your experiences of **consultation payment** for ECD care?  Note: Doctor only | - Experience/ clinic routine of charging patient for routine care (include challenges and how managed)?   - *Probes:*     - Charge a fixed amount for consultation (and core drug)?     - Charge separately for consultation and drugs (if any dispensed)? - Experience of adjusting the clinic charging for ECD care (include challenges and how managed)?   - *Probes:*     - Subsidized ECD follow-up consultation? |
| Would you kindly share your experiences on **identifying and referring development delays/ maternal depression**? | - Experiences of identifying and responding to the child development delays (include challenges & how managed)?   - *Probes:*     - Identifying development delays?     - Explaining mother? (possible reason and action)     - Convincing mother for specialist care need/up-take - Experiences of referring severe depression for specialist care (include challenges & how managed)?   - *Probes:*     - Mother denying     - Mother accepting diagnosis but refusing care     - Mother accessing specialist care     - Referred mother continuing ECD care at the clinic |

**Early Child Development Care Delivery through Private Clinics in Pakistan**

**Trial Process Evaluation**

**Tool-1: Interview Guidelines – Clinic Assistant**

| **Query** | **Prompts and Probes** |
| --- | --- |
| **Preparation for Care Delivery Process** | |
| Would you kindly share your experience regarding the **training** of **Early Child Development Care**? | - Experience of your getting trained on ECD care (include challenges and how managed)?   - *Probes:*     - Selected time and venue?     - Training contents – language, methods, duration, practical relevance etc?     - Trainer’s – knowledge and skill, facilitation ability,     - Arrangements – seating, cooling or heating, refreshment, time-keeping, house-keeping etc?     - Providing feedback on training conduct? |
| Would you kindly share your experiences of **engaging and working with community advocates**? | - Experience of engaging community advocates for ECD care promotion (include challenges and how managed)?   - *Probes:*     - Various types of community advocates?     - Referral arrangements for ECD care?     - Suggestion (if any) for future scaling of community advocates? |
| **Delivering the Intervention** | |
| Would you kindly share your experiences of **case examination and registration**? | - Experience of registering and examining child for ECD care (include challenges and how managed)?   - *Probes:*     - Educating mother to get her child registered for ECD care?     - Measuring the weight and length of child?     - Asking for quarterly clinic visit |
| Would you kindly share your experiences of **assessing and managing mothers’ depression**? | - Experience of screening and counselling mother for mental health (include challenges and how managed)?   - *Probes:*     - Administering PHQ-2? Mother responses?     - Administering counselling? - Experience of diagnosing and managing depression in mothers (include challenges and how managed)?   - *Probes:*     - Administering all PHQ-9 questions? Mother responses?     - Prescribing anti-depressant?     - Referring for specialist care (severe depression)? |
| Would you kindly share your experiences of **counselling** mother for **mental health**? | - Experiences of counselling mother for mental health (include challenges and how managed)?   - *Probes:*     - Arranging session e.g. clinic setting, time?     - Conducting a session – women participation, counselling contents, method, time-pressure, clinic care routine?     - Suggesting a follow-up session – technical, administrative, social, cost etc?     - Suggestion (if any) for future scaling of ECD counselling? |
| Would you kindly share your experiences of **maintaining records** for ECD care? | - Experience of keeping individual case records contrary to usual practice (include challenges and how managed)?   - *Probes*     - Logistics - time, space, clinic routine?     - potential benefit (e.g. PHC certification)?     - any risk (e.g. legal, tax) of maintaining case records? |
| Would you kindly share your experiences of mother-child **follow-up delayed visit**? | - Experience of contacting/convincing mother for follow-up visit (include challenges and how managed)?   - *Probes:*     - Identifying delayed visits     - Contacting mother or family member     - Convincing mother or family member |
| Would you kindly share your views on **mothers’ ability** to carry out ECD care at home? | - Experiences of mothers practicing ECD care ((include challenges and how managed)?   - *Probes:*     - Mother feedback     - Clinic staff response/ suggestion     - Suggestions for future |
| **Outcome Measurement of Intervention** | |
| Would you kindly share your experiences of the **clinic-based outcome measurements**? | - Experience of arranging measurement sessions at the clinic (include challenges and how managed)?   - *Probes:*     - Arranging the session? (arrange sitting/refreshment, invite/ remind mothers etc.)     - Providing environment for making queries to mothers and observing child responses?     - Conducting session (time management, clinic other activities)     - Suggestion, if any, for clinic-based measurements in future? |

**Early Child Development Care Delivery through Private Clinics in Pakistan**

**Trial Process Evaluation**

**Tool-1: Interview Guidelines – Research Staff**

| **Query** | **Prompts and Probes** |
| --- | --- |
| **Preparation for Care Delivery Process** | |
| Would you kindly share your experiences of **conducting** the **formative research** for ECD care? | - Experience of designing and conducting formative research (include challenges and how managed)?   - *Probes:*     - Scope and contents?     - Methods and tools?     - Participant selection and approach     - Facilitator – skills, language, gender, background     - Setting – venue, equipment, time, record taking |
| Would you kindly share your experiences of the **intervention development** process? | - Experience of identifying the key milestones and developing messages for ECD care (include challenges and how managed)?   - *Probes:*     - Selection of key development milestones     - Development of messages for each milestone?     - Development of pictorial sketches for the messages - Experience of assigning the care-tasks and outlining the care-delivery (include challenges and how managed)?   - *Probes:*     - Care-task assignment(contextualized)     - Care delivery – contents, and methods     - Requirements – setting, equipment, staff training - Experience of assessing training needs and developing training contents and methods (include challenges and how managed)?   - *Probes:*     - Staff training needs     - Training contents and methods (contextualized) |
| Would you kindly share your experience of the initial **survey** for the **selection** of your clinic in the new care delivery? | - Experiences of identifying poor urban localities; mapping and surveying; selecting private clinics (include challenges and how managed)?   - *Probes:*     - Poor urban localities – indicators, sources, use, limits?     - Mapping and surveying clinics – cooperation, data collected, data use,     - Selecting – transparency, clinic acceptance     - Future suggestions |
| Would you kindly share your experiences of **District Health Office engagement** in ECD care trial? | - Experiences of approaching and engaging district health office in ECD care trial (include challenges and how managed)?   - *Probes:*     - Engagement level (who) and modality (how, what information pack)     - Intervention and product development     - Clinic selection and support     - Implementation and monitoring     - Evaluation and dissemination     - Suggestions for better future engagement |
| Would you kindly share your experiences regarding the agreed **branding/promotion support**?  Note: Doctor only | - Experiences of developing and offering branding support (include challenges and how managed)?   - *Probes:*     - Developing structure (brand size, material)     - Developing contents for brand materials     - Implementing the clinic branding     - Perceived benefits and disadvantages     - Suggestions for future |
| Would you kindly share your experience regarding the **training** of **Early Child Development Care**? | - Experiences of arranging and conducting training of clinic staff (include challenges and how managed)?   - *Probes:*     - Arranging training events – venue, timing, facilitators, trainees     - Conducting training events – adhering to the design, duration and participation, facilitator-dependence (explanations), event/trainee assessment, post-training performance     - Suggestion for future ECD training? |
| Would you kindly share your experiences of **engaging and working with community advocates**? | - Experiences of selecting and engaging community advocates in ECD care promotion (include challenges and how managed)?   - *Probes:*     - Selecting, orienting and engaging various types of community advocates in ECD care promotion?     - Advocate referral of mother for ECD care at the clinic?     - Suggestion (if any) for future engagement of community advocates? |

| **Delivering the Intervention** | |
| --- | --- |
| Would you kindly share your experiences of **case examination and registration**? | - Experiences of clinic staff examining and registering newborns for ECD care (include challenges and how managed)?   - *Probes:*     - Weight and height examination (recording) practices     - Periodic calibration of equipment |
| Would you kindly share your experiences of **assessing and managing mothers’ depression**? | - Experiences of clinic staff assessing and managing mothers’ depression (include challenges and how managed)?   - *Probes:*     - Administering PHQ-2 and PHQ-9 at the clinic     - Counselling mothers for depression     - Indentifying and treating moderate depression     - Indentifying and referring severe depression |
| Would you kindly share your experiences of **maintaining records** for ECD care? | - Experiences of clinic staff keeping the case records at clinics (include challenges and how managed)?   - *Probes:*     - Completing individual case records and maintaining the quality of records |
| Would you kindly share your experiences of mother-child **follow-up delayed visit**? | - Experiences of clinic staff contacting and convincing mothers for follow-up ECD visit (include challenges and how managed)?   - *Probes:*     - Identifying delayed visits?     - Contacting the delayed mothers     - Convincing mothers to come for ECD care? |
| Would you kindly share your experiences of **consultation payment** for ECD care? | - Experiences of private clinics charging mothers for ECD consultation (include challenges and how managed)?   - *Probes:*     - Clinic motives and constraints?     - Mother responses and limitations |
| Would you kindly share your experiences on **identifying and referring development delays/ maternal depression**? | - Experiences of private clinic referring the severely delayed child and depressed mothers for specialist care (include challenges and how managed)?   - *Probes:*     - Referral arrangements – institution and referral process,     - Referral practices – clinic and mother perspectives |
| **Outcome Measurement of Intervention** | |
| Would you kindly share your experiences of the **clinic-based outcome measurements**? | - Experiences of arranging and conducting clinic-based outcome measurements (include challenges and how managed)?   - *Probes:*     - Designing and planning clinic-based measurement sessions?     - Arranging measurement sessions at respective clinics?     - Inviting mothers to measurement sessions?     - Sequencing various measurements e.g. child development, mother depression, social survey etc.     - Taking multiple measurements at each session     - Suggestion, if any, for clinic-based measurements in future? |
| Would you kindly share your experiences of **mothers’ observation responses** during outcome measurement? | - Experiences of getting mother’s observation on various child development measures (include challenges and how managed)?   - *Probes:*     - Making mother understand each query? (include asking if not clear)     - Getting and recording valid response to each query?     - Making assessor to follow the query protocol     - Managing the time for each set of queries? |
| Would you kindly share your experiences of the **on-site observation** during the outcome measurement? | - Experiences of getting assessor’s observation on various child development measures (include challenges and how managed)?   - *Probes:*     - Maintaining environment for child comfort (for on-site observation)?     - Making assessor tell mother what act to ask the child to perform?     - Promoting child carryout various acts?     - Recording valid observation on each act?     - Managing discrepancy in mother and onsite observation?     - Managing time for each set of acts? |

**Early Child Development Care Delivery through Private Clinics in Pakistan**

**Trial Process Evaluation**

**Tool-1: Interview Guidelines – Client (Mother)**

| **Query** | **Prompts and Probes** |
| --- | --- |
| **Preparation for Care Delivery Process** | |
| Would you kindly share your experiences of **District Health Office engagement** in care delivery? | - Does public endorsement of a private clinic make any difference for a client? If yes; what and why?   - *Probes:*     - Cost of care?     - Quality of care?     - Public accountability? |
| Would you kindly share experiences of contacting a **branded clinic** for ECD care? | - Your experience of contacting a branded clinic for ECD care?   - *Probes:*     - Noticed the branding? why (topic, presentation, other)?     - Role in your decision to attend ECD care or the clinic?     - Information that was found more relevant and useful?     - Suggestion to improve branding contents and/ or display? |
| Would you kindly share experiences of your getting **informed/ referred** for ECD care? | - Does the community advocate engagement influences mother’s uptake of ECD care?   - *Probes:*     - What influence? and why?     - Suggestions for future engagement of community advocates (type of advocate, ECD knowledge, referral) |
| **Delivering the Intervention** | |
| Would you kindly share experiences of getting your child **examined** and **registered** for ECD care? | - Experience of getting the first ECD care examination and registration at the clinic?   - *Probes:*     - Who and how explained her the need for ECD care?     - Why did you get your child registered?     - Any concern about her child getting measured?     - Suggestion for future ECD care? |
| Would you kindly share experiences of your getting **assessed** and **treated** for depression? | - Experience of getting screened and counselled for mental health (include challenges and how managed)?   - *Probes:*     - Assessment for depression (i.e. administering of PHQ-2 and PHQ-9) – understand and respond questions?     - Getting labelled and prescribed drugs (for depression)     - Disclosing the diagnosis and taking anti-depressant drugs?     - Getting referred for specialist consultation (depression care)? |
| Would you kindly share experiences of your getting **counselled** for mental health? | - Experience of getting counselled for mental health?   - *Probes:*     - Clinic setting for counselling?     - counselling contents i.e. relevant, practical, acceptable, understandable,     - Methods i.e. pictorial tool assisted, interactive,     - Need for quarterly follow-up sessions i.e. worth for a repeat visit - time, cost, effort     - Suggestion (if any) for future scaling of ECD counselling? |
| Would you kindly share experience of the clinic **maintaining** your (& child) **records** for ECD care? | - Experience of the clinic keeping your records i.e. child and mother health (include challenges and how managed)?   - *Probes*     - Clinic practices     - Concerns, if any     - Suggestion for future scaling |
| Would you kindly share experiences of your visiting clinic for continued ECD care (also getting contacted, by clinic staff, for a reminder to visit)? | - Experience of your visiting the clinic every quarter for continued ECD care (include challenges and how managed)?   - *Probes*     - Social and cultural     - Financial     - Others - Experience of your getting contacted, by clinic staff, for a delayed ECD care follow-up visit   - *Probes*     - Access     - Social and cultural     - Literacy and others |
| Would you kindly share experiences of your paying a **fee** for child development **consultation**? | - Experience of your requiring a clinic visit when child is not ill (include challenges and how managed)? - Experiences of your paying for ECD consultation (include challenges and how managed)?   - *Probes:*     - Clinic facilitation - e.g. subsidy, exempt queue etc.     - Mother response – e.g. acceptable; helpful; |
| Would you kindly share experiences of your **practicing ECD** care at home? | - Experiences of your practicing ECD care activities at home (include challenges and how managed)?   - *Probes:*     - Acceptable and feasible – time, energy, skills, environment     - Useful – help child development     - Suggestions for future |
| Would you kindly share experiences of your getting **referred** for a delay in child development and/or maternal depression? | - Experiences of your getting referred for specialist consultation (include challenges and how managed)?   - *Probes:*     - Getting labelled as severely ill - needing referral care?     - Accessing the referral care (e.g. distance, timing, cost, repeated visits etc.)     - Availing the referral care (e.g. specialist consultation, added investigations, getting problem solved)? |
| **Outcome Measurement of Intervention** | |
| Would you kindly share experiences of getting your child assessed at the **clinic** (at completion of one year)? | - Experience of getting your (one-year old) child assessed at the respective clinic (include challenges and how managed)?   - *Probes:*     - Attending the clinic-based assessment – date/ time, venue, information     - Getting your child development assessed – setting, time required, arrangements, assessment team,     - Getting your mental health assessed     - Suggestion for clinic-based assessments in future? |
| Would you kindly share experiences of your **observation responses** during the child development assessment? | - Experience of sharing your observations on child development (include challenges and how managed)?   - *Probes:*     - Understanding the queries? (include asking if unclear)     - Responding to the queries?     - Getting informed about child development progress?     - Assessor’s recording the measurements     - Any coercion or influence to modify your response? |
| Would you kindly share experiences of assessor making **on-site observation** during the child development assessment? | - Experience of assessor making an on-site observations of your child development (include challenges and how managed)?   - *Probes:*     - Understanding the asked child acts?     - Making child responding to the asked acts?     - Getting explained about child non-performance and/or any observation discrepancy? |
