# Supplementary material for: Integrating maternal depression care at primary private clinics in low-income settings in Pakistan: A secondary analysis
Source: Front Glob Womens Health. 2023 Apr 6;4:1091485. doi: 10.3389/fgwh.2023.1091485 (PMC10117980; doi:10.3389/fgwh.2023.1091485)
Supplement: Supplementary file 2 [file Table2.docx]

**Appendix 2**

**Predicted proportional odds from random-intercept ordered logistic model estimating improvement in PHQ-9 depression category for mothers at private clinics (N=1,957, Intervention= 1,037, Control= 920)***

|  | Intervention (95% CI) | Control (95% CI) |
| --- | --- | --- |
| Crude Model | | |
| Positive^a^ | 0.23 (0.20, 0.26) p=0.00 | 0.03 (0.02, 0.04) p=0.00 |
| No change^b^ | 0.76 (0.73, 0.79) p=0.00 | 0.92 (0.91, 0.94) p=0.00 |
| Negative^c^ | 0.005 (0.003, 0.007) p=0.00 | 0.05 (0.03, 0.06) p=0.00 |
| Log likelihood | -882.51 | |
| Adjusted Model | | |
| Positive^a^ | 0.27 (0.13, 0.40) p=0.00 | 0.023 (0.004, 0.04) p=0.018 |
| No change^b^ | 0.73 (0.60, 0.86) p=0.00 | 0.92 (0.89, 0.95) p=0.00 |
| Negative^c^ | 0.003 (0.001, 0.007) p=0.016 | 0.06 (0.01, 0.98) p=0.012 |
| Log likelihood | -870.41 | |

*Proportional odds were calculated using margins from a random intercept ordered logistic regression estimates and corresponding thresholds for the three categories within ΔPHQ-9 category variable. ^a^Represents an improvement in PHQ-9 measured depression category, into a lesser depression category or no depression compared to baseline. ^b^Represents no change in PHQ-9 depression category between baseline and endpoint measurement. ^c^Represents a worsening of PHQ-9 depression category into a higher depression category at endpoint compared to baseline.
